# Pattern Genes Suggest Functional Connectivity of Organs

**DOI:** 10.1038/srep26501

**Published:** 2016-05-26

**Authors:** Yangmei Qin, Jianbo Pan, Meichun Cai, Lixia Yao, Zhiliang Ji

**Affiliations:** 1 State Key Laboratory of Cellular Stress Biology, School of Life Sciences, Xiamen University, Xiamen, Fujian, 361102, P. R. China; 2 Department of Chemical Biology, College of Chemistry and Chemical Engineering, The Key Laboratory for Chemical Biology of Fujian Province, Xiamen University, Xiamen, Fujian, 361005, P. R. China; 3 Department of Software and Information Systems, University of North Carolina at Charlotte, North Carolina, 28105, USA

## Abstract

Human organ, as the basic structural and functional unit in human body, is made of a large community of different cell types that organically bound together. Each organ usually exerts highly specified physiological function; while several related organs work smartly together to perform complicated body functions. In this study, we present a computational effort to understand the roles of genes in building functional connection between organs. More specifically, we mined multiple transcriptome datasets sampled from 36 human organs and tissues, and quantitatively identified 3,149 genes whose expressions showed consensus modularly patterns: specific to one organ/tissue, selectively expressed in several functionally related tissues and ubiquitously expressed. These pattern genes imply intrinsic connections between organs. According to the expression abundance of the 766 selective genes, we consistently cluster the 36 human organs/tissues into seven functional groups: adipose & gland, brain, muscle, immune, metabolism, mucoid and nerve conduction. The organs and tissues in each group either work together to form organ systems or coordinate to perform particular body functions. The particular roles of specific genes and selective genes suggest that they could not only be used to mechanistically explore organ functions, but also be designed for selective biomarkers and therapeutic targets.

Human organ is a community of cells of different types. The cells are assembled organically into either main tissue (parenchyma) or sporadic tissues (stoma) to carry out a particular physiological function. Theoretically, the exact functions that an organ performs are subject to its constituted cells, specialized during development and are accompanied with a bundle of cellular processes including intracellular interactions, cell-cell contacts, and extracellular matrix (ECM) modeling mediated by grow factors and hormones^1^. Unfortunately, the exact molecular mechanisms underlying these processes are largely unknown and hard to be fully characterized using existing technologies. To large extent, we only know that organ functions are the reflection of biochemical reactions of proteins, which are the products of gene transcription and translation in the component cells. Hence, the differential gene expression patterns across organs, represented by their main tissues in many cases, may cast light on what leads to specific organ functions.

So far, three major modularly expressed gene groups (or namely pattern genes) with spatial expression patterns across organs/tissues have been identified, namely housekeeping genes, selective and specific genes. Housekeeping genes express ubiquitously and evenly (in strict sense) in organs (or their main tissues) to maintain basal cellular functions^2^. Deficiencies or non-synonymous mutations in housekeeping genes will likely lead to diseases^3^. Selective genes have enriched expressions in several organs, tissues or cell types^4^. In the extreme cases, if a gene expresses only in one organ, it becomes specific. Previously, the tissue-specific or selective genes have been linked to different biomedical aspects of transcriptional regulation^5^,^6^, epigenetic modification^7^ differentiation^8^, pathogenesis^9^,^10^, biomarker design^11^ and evolution^12^. These efforts reveal limited characteristics of tissue and organ differentiation due to small data size or lack of rigorous quantitative analysis. Importantly, few of these studies have investigated the molecular mechanisms underlying organ crosstalk, which helps assembling different organs into organ system to perform complicated body functions. Moreover, when closely looking into those existing studies, we found the inconsistent assignments of housekeeping, selective and specific genes. Many of the studies may have “falsely” identified pattern genes using either low throughput technologies like RT-PCR or experimental results from limited tissue samples. For example, the conventional housekeeping genes such as GAPDH, PPIA, and ACTB were proved to be not ubiquitous across tissues^13^,^14^,^15^. Not to speak of the specific genes, the specific genes identified by different analysis methods^16^,^17^,^18^,^19^,^20^ from the same high throughput dataset are largely varied (Fig. 1a).

Therefore in this study, we are motivated to identify pattern genes from 8 carefully selected human transcriptome datasets (6 microarray datasets and 2 next generation sequencing (NGS) datasets) that represent 36 organs/tissues and multiple cell types under normal physiological conditions (see Supplementary Table S1). We want to use these pattern genes to heuristically answer questions like what decides organ-specific functions? What mediates the functional connection between organs? And how do organs work together to realize body functions?

## Results

### Consensus Identification of Pattern Genes in Various Organs and Tissues

**Figure 1:**
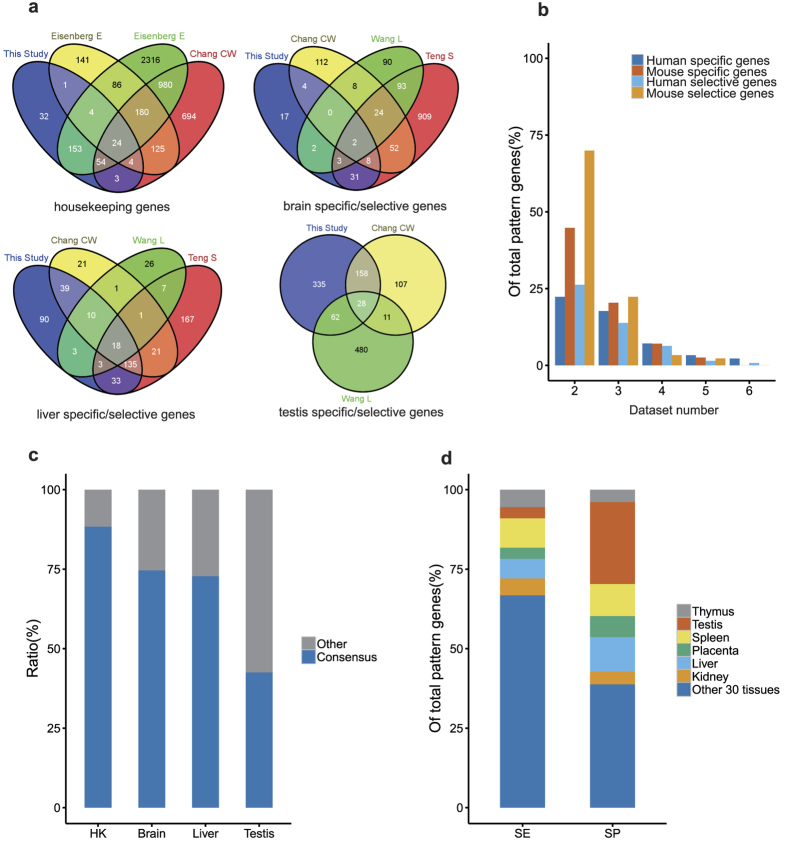
Overview of Human Tissue Pattern Genes. (**a**) Comparison of pattern genes identified in this study and prior studies. (**b**) Comparison with *Mus musculus* pattern genes identified from five transcriptome datasets. (**c**) Ratio of consensus housekeeping genes (HK) that verified by at least one other study in all selective organs, brain, liver and testis. (**d**) The distribution of selective genes (SE) and specific genes (SP) in organs.

The six selected datasets represent 36 distinct organ/tissue samples, among which six organs/tissues and 3,949 genes are common to all of them. After consensus evaluation (see Methods), we identified 2,108 specific genes, 766 selective genes and 275 housekeeping genes in the 36 distinct organ or tissue samples (Table 1). Of these genes, 95 consistently occur to all the six common organ/tissue samples, including 84 specific genes and 11 selective genes (corresponding to 13 selective gene-organ pairs). Additionally, we did the same analysis for five separate datasets of *Mus musculus*, the most often used animal model, and found that 8 out of 76 organs and 5,072 out of 22,576 genes were common to all five selected mouse datasets, among which 245 pattern genes were consistently identified (Fig. 1b). The similar level of discrepancy for pattern gene assignments in heterogeneous datasets was probably mostly caused by sampling variation. On top of that, different pattern gene identification methods may also account for the unreliability and inconsistence^19^. We compared the housekeeping genes and specific genes identified in this study with those identified in five prior studies^16^,^17^,^18^,^19^,^20^ and found only a very small portion of the common pattern genes (Fig. 1a). Comparatively, 88.36% of housekeeping genes, 72.81% of liver-specific genes, 74.63% of brain-specific genes, and 42.54% of testis-specific genes identified in this study were validated by one or more prior studies (Fig. 1c).

The majority of the specific pattern genes are found mostly abundant in human organs like testis, liver, and brain that perform highly specialized functions (Fig. 1d). For instance, about 44.8% (519) of specific genes are identified in testis and about 11.9% are found in liver. Comparatively, 206 out of 519 testis-specific homologous genes were also found to be specific in mouse (out of 814 testis-specific mouse genes). Similar distribution occurred in liver. Such results caution us on using animal models for human research, if it involves these organ-specific body functions.

### Roles of Pattern Genes in Organ Functions

Our gene ontology enrichment analyses on consensus pattern genes show the housekeeping genes usually encode various binding proteins, which reside at the intracellular membrane of organelles and are involved in cellular metabolisms of macromolecules and organic substances (Supplementary Fig. S1a). This result is partially consistent with prior studies, where housekeeping genes were found mainly involved in translation^19^,^21^,^22^, oxidative phosphorylation^19^,^21^, cell signaling/communication and structure/motility^22^. Considering their crucial roles in cell life, any deficiency or defect of housekeeping genes is susceptible to diseases in whole body^3^. By contrast, the specific genes show no obvious molecular function or cellular component propensity, except that about half of the specific genes are involved in single organism processes and response to stimulus (Supplementary Fig. S1b). Many selective genes encode extracellular membrane proteins. They often serve as transporters or receptors, participating in multiple key cellular processes such as cell communication, signal transduction, response to stimulus and structure development (Supplementary Fig. S1c).

To reveal the impact of pattern genes on organ functions, we further annotated the organs according to their specific genes. As shown in the Supplementary Table S2, minor or novel organ functions were discovered beyond our common knowledge of organs. For instance, liver plays important roles in detoxification, drug metabolism, lipid metabolic process and synthesis, and metabolism of bile acid. Other than these well-known functions, we also observed liver’s minor roles in immune response and in hormonal regulation. Some of these minor liver functions have been noticed^23^ but not well illustrated. Besides, the organ-specific genes are crucial not only for executing particular organ function, but also for maintaining organ structures. Of 183 genes related to tissue structure in human genome, 48 are specific or selective genes and none is housekeeping gene. Interestingly, of the rest 135 genes, 95 genes intend to enrich in several tissues/organs and only 12 genes ubiquitously express in all tissues.

In many cases, the organ-specific expressions are the combinational outcome of cell-specific expressions. However, the cell type-specific expression does not always suggest tissue-specific expression (Supplementary Table S3). Especially, the relation between tissues and cell components are often promiscuous. Other factors like abundance of component cells, cell development stages, and physiological environments and so on play roles. For instance, PHD finger protein 13 (SPOC1) is a testis-specific gene exclusively localized in germ cell spermatogonia^24^, which made it a good biomarker in monitoring spermatogenesis but not in other testis-related events.

### Selective Genes Mediate Functional Connectivity of Human Organs

We carried out clustering analyses on the six selected datasets using the method described in previous work^25^, but yielding completely different topologies of organ similarity (Supplementary Fig. S2a). Even after excluding the dataset discrepancy caused by the different sampling, the six common organs did not cluster consistently (Supplementary Fig. S2b). These results indicate that genome wide gene expression data may not be applicable for constructing functional connectivity between organs due to big noise. Principle component analysis (PCA) did mildly improve the organ topology consistence (Supplementary Fig. S2c,d); but it is not consensus enough to build the solid organ connectivity.

**Figure 2:**
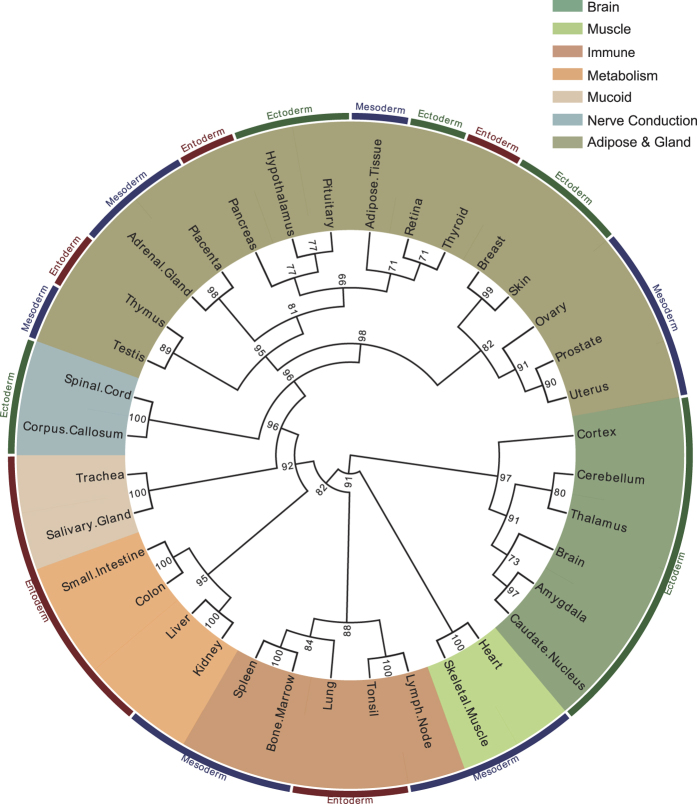
The human organ connectivity map. The 36 human organs were grouped, using the Ward. D method, into seven clusters according to the correlation coefficients between organs determined upon their commonality of selective genes. These clusters were further annotated as brain, immune, muscle, metabolism, mucoid, nerve conduction and adipose&&gland, respectively. The development origins of organs were also labeled outside the map circle in red, blue and green, corresponding to endoderm, mesoderm and ectoderm respectively. The values on the edges of the clusters stand for AU (Approximately Unbiased) p-values, indicating how many times the clustering were supported in 100 repeats.

So what exactly determines the connectivity between organs? To answer this question, we further clustered organs based on consensus selective genes identified in this study. The results were illustrated in Supplementary Fig. S2e,f. It seemed that the selective genes could consistently repeat the organ topology. Upon the distribution similarity of 766 consensus selective genes over 36 human organs/tissues, we constructed an organ connectivity map (Fig. 2). In this map, the 36 human organs were grouped into seven major clusters. These clusters were then annotated according to their commonality in organ functions, which are adipose & gland, brain, immune, metabolism, mucoid, muscle, and nerve conduction respectively. Organs/tissues in each of these clusters and their sub-clusters are likely to share some common physiological functions (Supplementary Table S4), for example, both liver and kidney are involved in endogenous and exogenous compound metabolism. The organ connectivity map helps us understand organ crosstalk and infer novel organ cooperation. For instance, thyroid hormones’ regulating cone opsin expression in the retina^26^ and adiponectin expression in adipose tissue^27^ may partially explain why thyriod, retinal and adipose tissue were grouped into the same sub-cluster in the map. More interestingly, prostate, uterus and ovary, were assigned into the same sub-cluster with mild confidence. This assignment is supported by previous finding that prostatic tissue exits in the uterine cervix^28^ and a benign cystic teratoma of ovary^29^, although the mechanism still need to be uncovered.

**Figure 3:**
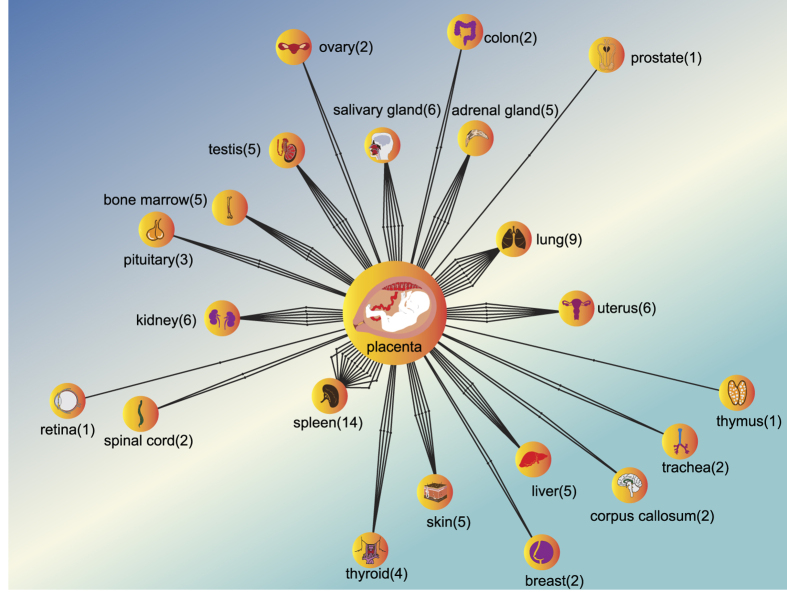
The placenta-centralized organ connectivity map. This map was constructed upon their mutual selective genes, the more selective genes shared between tissues and placenta, the closer they are. Spleen (14), lung (9), kidney (6), salivary gland (6) and uterus (6) are the top five organs that might functionally connect with placenta. Author: freedesignfile, http://all-free-download.com/.

In addition, we built a placenta-centralized organ connectivity map, which connects the placenta with many other organs based on the mutual selective genes (Fig. 3). In this map, spleen, lung, kidney, salivary gland, and uterus are the top five organs that have mostly close connection with placenta. We compared the organ functions between the placenta and other organs, linking them with the gene functions (Supplementary Table S5). It is a natural thought that the enriched gene expression may more or less contribute to the organ functions. In this sense, the placenta could be much more versatile than it is commonly known in nutrient uptake, waste elimination, gas exchange and neuroendocrine. Based on these data, we propose that the placenta may serve as a tissue/organ adaptor, performing simplified but essential organ functions that are usually exerted by other fully developed human organs like lung, liver, kidney and so on. For instance, glycoprotein hormones, alpha polypeptide (CGA), chorionic somatomammotropin hormone 2 (CSH2), and delta-like 1 homolog (DLK1) were found selectively expressed in adult pituitary and placenta. During the pregnancy, these three genes encode glycoprotein hormones and perform pituitary functions like regulation of steroid hormone synthesis (CGA), growth control (CSH2) and neuroendocrine differentiation (DLK1) to guarantee fetal normal growth.

### Selective Gene Expressions Likely Infer Organ Development Path

Of 2,784 consensus specific/selective genes identified in this study, 644 genes (or 23.13%) are associated with tissue or organ development, comparing to 2,985 out of 18,209 (or 16.4%) genes in human genome background. We labeled the organ connectivity map according to the organ-derived germ layers during the organogenesis (Fig. 2). It was observed that organs/tissues in the same cluster or sub-cluster often come from the same blastoderm. For instance, the neuron system (both brain and nerve conduction clusters) develops from the ectoderm; organs in the muscle cluster develop from the mesoderm; organs in the mucoid cluster develop from the entoderm. Unlike the other organ clusters, the adipose cluster, the largest organ cluster in this map, does not show coincident development origin. However, many organs/tissues in the sub-clusters have same development origin from ectoderm, mesoderm or endoderm. The coincidence of organ origin in the cluster or sub-cluster suggests that tissue-selective genes can also serve as an indicator in understanding of organ origin.

## Discussions

What decides organ functions? How does it relate to the different gene expression profiles? To address these questions, we identified a number of consensus pattern genes in this study and upon which we carried out a series of computational analyses (Fig. 1 & Supplementary Fig. S1, Supplementary Tables). We find that the majority of housekeeping genes are involved in maintaining basal cellular processes like intracellular metabolism. By contrast, the specific genes intend to play various roles in extracellular transportation and exert specific functions in organ development. It is likely that the specific genes differentiate the organs from each other by responding to ion-like stimulus outside the cell. Moreover, we also find that the selective genes may suggest the functional commonality between organs. Although, multiple aspects of our studies illustrate different functional propensities of pattern genes, only a few of these findings have enough direct molecular evidences via literature surveillance. Further experimental validations are thus desired.

Furthermore, Our genome wide similarity analyses on six independent datasets verify prior study that differential gene expression patterns are unreliable to infer tissue functions^25^ even after principle component analysis (Supplementary Fig. S2). Instead, we propose a universal mechanism that the selective genes likely play a messenger role between whole body organs rather than some restricted organ functions. Based on the selective genes, heterogeneous datasets can yield a consensus topology of organ relationship (Supplementary Fig. S2). As well, we drew a human organ connectivity map upon selective genes (Fig. 2). In this map, organs in the same clusters or sub-clusters tend to integrate and form higher organ system like central and peripheral nerve system. Some organs in the same cluster may join together to perform particular physiological functions; for examples, both liver and kidney are involved in compound metabolism, heart and skeletal muscle form similar organ structure (Supplementary Table S4).

Then, how do organs work together to realize body functions? So far, we know that many, 325 out of 766 (or 42.4%), organ selective genes are involved in cellular communication. These selective genes may partially answer for the organ cooperation. Moreover, the common cell compositions of different tissues, to some extent, can also explain the functional connectivity between organs. We also acknowledge that there still exist many uncertainties to be addressed: Does the same cell type play exactly the same roles in different tissues/organs? What is the mechanism behind the organs of different systems for a basic body function?

Nevertheless, the specific biological characteristics of these organ pattern genes, such as housekeeping genes in metabolism, specific genes in organ development and selective genes in organ crosstalk, suggest possible novel biomarkers or therapeutic targets for precise and efficient prognosis and treatment of complicated diseases^21^,^30^.

## Material and Methods

### The High-Throughput Datasets

We selected eight high-throughput datasets, including six microarray datasets and two next-generation sequencing (NGS) datasets (Supplementary Table S1) from the public repositories GNF BioGPS and NCBI GEO. The datasets were chosen according to the following criteria: (1) consisting of a large number of genes, (2) having samples obtained from at least five distinct human tissues or organs, and (3) representing normal physiological states. We then removed incomplete or defected profiles and used mean values to represent gene expression levels if there are multiple replicates in a dataset.

### Identification of Consensus Patterns for Genes

The organ/tissue expression patterns for housekeeping, specific and selective genes were identified from the eight datasets separately using the statistical method described in our previous work^15^ (as well as in the Supplementary Method) and the conventional proportion method. To reconcile the discrepancy in pattern gene assignment upon these heterogeneous datasets, we introduce a parameter called the occurrence (O):


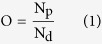


where N_p_ is the number of times of a given gene is assigned to express in a given tissue; and N_d_ is the number of datasets that contain both the given gene and the organ or tissue sample (especially for specific/selective gene-tissue pairs). Based on this occurrence measure, we define a consensus gene should meet two criteria: (1) N_d_ ≥ 2 and (2) the occurrence O ≥ 0.5. For tissue selective genes, an additional criterion that gene expresses in at least two organs or tissues must meet. The specific gene is the extreme case of the selective gene, where a specific gene only occurs in one organ or tissue, and as for housekeeping gene, which are expressed ubiquitously across tissues under all physiological conditions and developmental stages. We only included genes with the consensus expression patterns in the next steps of analyses.

### Construction of Organ-Specific Connectivity Map

We built a binary matrix for all the genes with consensus expression patterns as following: when a gene is selectively expressed in an organ or tissue, the cross element of this gene column and organ row in the matrix is set to 1, otherwise it is set to 0. In this way, we created a matrix of 36 rows – representing distinct organ or tissue samples and 766 columns – representing the total number of distinct genes. Then we used the R package “pvclust”^31^ to run clustering analysis by calculating the organs’ similarity in terms of the expression profile of selective genes. The parameters for the pvclust package were set to method.hclust = “ward.D”, method.dist = “cor”. Not the initial value was given when running package “Pvclust”. As well, the hierarchical clustering was repeated 100 times with multi-scale bootstrap re-sampling. The bootstrap value indicates how strong the clustering is supported.

## Additional Information

**How to cite this article**: Qin, Y. *et al.* Pattern Genes Suggest Functional Connectivity of Organs. *Sci. Rep.* **6**, 26501; doi: 10.1038/srep26501 (2016).

## Supplementary Material

Supplementary Information

Supplementary Table S2

Supplementary Table S4

Supplementary Table S5

## Figures and Tables

**Table 1:** Distribution of pattern genes across datasets.

Dataset number	6	≥5	≥4	≥3	≥2
Gene	3,949	7,701(3,752)	11,002(3,301)	15,687(4,685)	18,999(3,312)
Tissue	6	13(7)	21(8)	29(8)	36(7)
Selective gene-organ pair*	13	70(57)	279(209)	940(661)	1,811(871)
Specific gene	84	203(119)	442(239)	1,274(832)	2,018(744)
Housekeeping gene	0	1	21(20)	102(81)	275(173)

The number in the bracket stands for the exact number of pattern genes in corresponding dataset number.

^*^A selective gene-organ pair stands for the combination of a gene and an organ/tissue. For example, gene ABCG5 selectively expresses in liver and small intestine, which will generate two gene-organ pairs: ABCG5-liver and ABCG5-small intestine.
