# Supplementary material for: Pattern Genes Suggest Functional Connectivity of Organs
Source: Sci Rep. 2016 May 26;6:26501. doi: 10.1038/srep26501 (PMC4880915; doi:10.1038/srep26501)
Supplement: Supplementary Information [file srep26501-s1.pdf]

SUPPLEMENTARY INFORMATION

Pattern Genes Suggest Functional Connectivity of Organs.

Yangmei Qin[1,+], Jianbo Pan[2,+], Meichun Cai[1], Lixia Yao[3], Zhiliang Ji[1, 2, *]

[1] State Key Laboratory of Cellular Stress Biology, School of Life Sciences, Xiamen University, Xiamen, Fujian, 361102, P R China

[2] Department of Chemical Biology, College of Chemistry and Chemical Engineering, The Key Laboratory for Chemical Biology of Fujian Province, Xiamen University, Xiamen, Fujian, 361005, P R China

[3] Department of Software and Information Systems, University of North Carolina at Charlotte, North Carolina, 28105, USA

[+]These authors have equal contribution

*To whom it may correspond to:

Zhiliang Ji,

Tel: 86-0592-2182897;

Fax: 86-0592-2182897;

Email: appo@xmu.edu.cn

Supplementary Method

Pattern genes are identified from eight human transcriptome datasets using "SPM" method, which also described in our previous work[1]. "SPM" method includes three statistical parameters: SPM, DPM and CTM. They are described briefly below:

First, each gene expression profile is transformed into a vector *X*:

$$X = (x_1, x_2, ..., x_i, ..., x_{n-1}, x_n)$$ [*n* is the number of samples in the profile]

Similarly, a vector X_i can be generated to represent the gene expression in sample (condition) *i*:

$$X_i = (0, 0, ..., x_i, ..., 0, 0)$$

SPM : Specificity Measure

SPM is the cosine value of the intersection angle θ between vectors X_i and X in high dimensional feature space, which measures the specificity of a gene's expression in a designated sample.

$$SPM = \cos\theta = \frac{X_i \cdot X}{|X_i| \cdot |X|}$$

So a gene expression profile (*X*) can be converted to a corresponding SPM profile (X_{SPM}):

$$X_{SPM} = (SPM_1, SPM_2, ..., SPM_i, ..., SPM_{n-1}, SPM_n)$$

DPM : Dispersion Measure

DPM is the standard deviation in unitary form based on the transformed SPM profile:

$$DPM = \sqrt{\frac{\sum_{i=1}^{n} (SPM_i - \overline{SPM})^2}{n-1}} \cdot \sqrt{n}$$ [$\overline{SPM}$ is the mean value of *SPM*s in a gene expression profile]

CTM : Contribution Measure

CTM measures the enrichment of gene expression levels in several samples:

$$CTM_k = \sqrt{\sum_{i=1}^{k} SPM_i^2}$$ [*k* is the number of selected samples, 2≤k≤6]

Pattern genes can be defined and evaluated using the three statistical parameters defined above (SPM, DPM, and CTM) alone or in combination:

Housekeeping gene : DPM < 0.3

Selective gene : $2 \le k \le 6$, $SPM_{i\ (1\ to\ k)} > 0.3$, and $CTM_k > 0.9$

Specific gene : SPM > 0.9

References

1.Pan, J. B. et al. PaGenBase: a pattern gene database for the global and dynamic understanding of gene function. PloS one 8, e80747, doi:10.1371/journal.pone.0080747 (2013).

Supplementary Figure S1. Gene ontology enrichment of pattern genes.

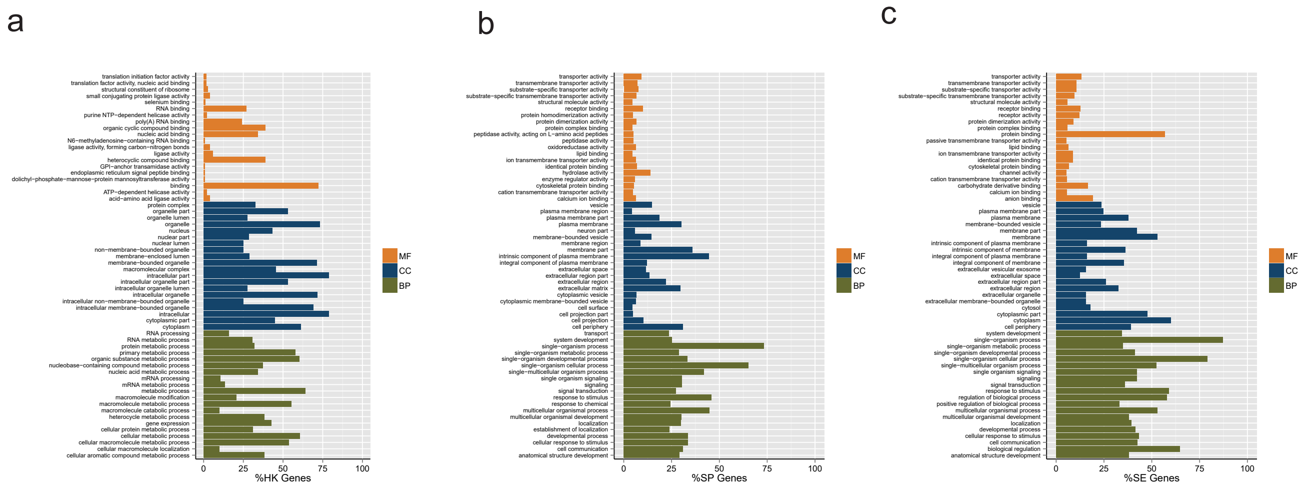

Supplementary Figure S1. Gene ontology enrichment of pattern genes.
(a) Housekeeping genes (HK). (b) Specific genes (SP). (c) Selective genes (SE). The Go Term were sorted in alphabetical order.

Supplementary Figure S2. Organ clustering on six selective datasets.

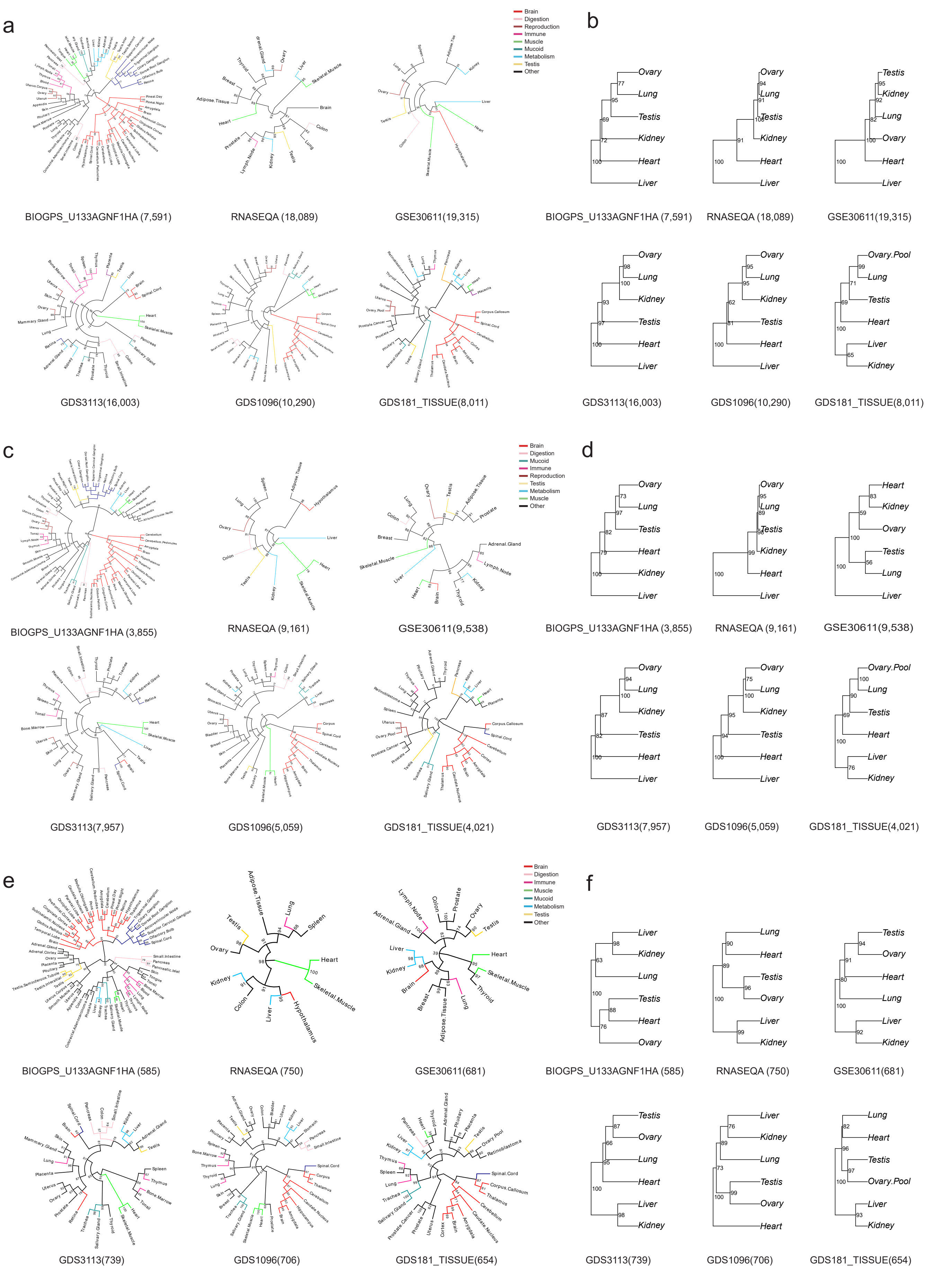

Supplementary Figure S2. Organ clustering on six selective datasets.
The dataset name and the number of genes (in brackets) that used to make organ cluster were given under each cluster. (a), (c) and (e) used all organs/tissues in the datasets for clustering. (b), (d) and (f) used 6 common organs (heart, kidney, liver, lung, ovary and testis) for clustering. (a) and (b) were clustered upon genome wide gene co-expression. (c) and (d) were clustered upon feature gene co-expression after principle component analysis (PCA). (e) and (f) were clustered upon selective genes.

Supplementary Table S1. Datasets involved in this study.

Dataset	PubMed ID	Title	Type
BIOGPS_U133AGNF1HA	15075390	Tissues of various types	Tissue
GDS1096	15950434	Normal tissues of various types	Tissue
GDS181_TISSUE	11904358	Large-scale analysis of the human transcriptome (HG-U95A)	Tissue
GSE30611	N.A.	[E-MTAB-513] Illumina Human Body Map 2.0 Project	Tissue
GDS3113	19014478	Various normal tissues	Tissue
RNASEQ	22345621	RNA-Seq gene expression profiles of 15 tissues	Tissue
GDS1835	15113400	Various cell lines and Universal Reference RNA (II)	Cell
BIOGPS_U133AGNF1HB	15075390	Cells of various types	Cell

Supplementary Table S3. Identification of cell-specific genes and its comparison with organ-specific genes.

Cell Type	Cell-specific Gene	Organ-specific Gene	Dataset
Brain Cell	65	0	GDS1835
Breast cell	53	0	GDS1835
Liver Cell	77	24	GDS1835
Testis Cell	22	1	GDS1835
Testis Germ Cell	77	25	BIOGPS_U133AGNF1HB
Testis Leydig Cell	9	4	BIOGPS_U133AGNF1HB
Testis Germ-Leydig Cell	195	81	BIOGPS_U133AGNF1HB
